# Kinetics of dissolution and computational modeling of calcium oxalate monohydrate crystals in the presence of aqueous coffee bioactive extract compounds

**DOI:** 10.1038/s41598-026-40198-y

**Published:** 2026-03-23

**Authors:** Eman T. Khattab, Naema S. Yehia, Mahmoud A. S. Sakr, Hesham R. El-Seedi, Heba A. El-Shekheby

**Affiliations:** 1 Chemistry Department, Faculty of Science, Menoufia University, Shebin El- Kom, Egypt; 2 Chemistry Department, Center of Basic Science, Misr University for Science and Technology (MUST), Giza, P.O. 77 Egypt; 3 Department of Chemistry, Faculty of Science, Islamic University of Madinah, Madinah, 42351 Saudi Arabia

**Keywords:** Dissolution, Urolithiasis, Calcium oxalate monohydrate, Coffee extract, Chemistry, Materials science

## Abstract

Urolithiasis, predominantly caused by calcium oxalate crystallization and dissolution, remains a major clinical challenge. This study explored the effect of coffee extract bioactive metabolites on synthetic COM crystals dissolution through combined experimental and computational approaches. LC–LTQ–MS/MS and NMR profiling identified chlorogenic acids, quinic acid derivatives, and caffeine as the principal constituents. Constant-composition dissolution method assays showed a concentration-dependent inhibition of COM dissolution, consistent with a film surface–controlled mechanism. Langmuir adsorption analysis revealed strong surface affinity (*K*_L_ = 2.274 × 10^4^ dm³ mol^−^¹, Δ*G*_ads_ = − 36.23 kJ mol^−^¹). Density functional theory (DFT) calculations highlighted caffeine as a key inhibitor, forming stable hydrogen bonding and van der Waals interactions with COM (*E*_a_ for adsorption = − 0.273 eV). FTIR, SEM, EDX, and XRD analyses confirmed physical adsorption without altering crystal structure. These findings suggest that coffee metabolites act as natural modulators of COM behavior, offering promising insights into urolithiasis prevention.

**Supplementary Information:**

The online version contains supplementary material available at 10.1038/s41598-026-40198-y.

## Introduction

Urolithiasis is a pathology characterized by the formation of stones in the kidneys or urinary tract. The Global epidemiological profile appears consistent. Prevalence ranges from about 1% to 20% depending on the region^1,3^. In 2021, about 106 million new cases of urolithiasis were diagnosed worldwide^2,3^. The age-standardized incidence rates are around 1240 per 100,000 population (or 1.24%), higher in men than in women. Overall prevalence estimates ranged between 1% and 20% across different populations^3,4^. Stones are composed mainly of calcium oxalate, and they reappear in over half of the patients^5^. Several studies have shown that calcium oxalate (CaOx) accounts for approximately 70% of the main chemical species of stones worldwide^6^. There are three distinct crystalline forms of kidney stones, which are poorly soluble in water or alkaline solutions^7^. The most prevalent and most stable form at body temperature (37 °С) is the monohydrate (COM, whewellite), which is usually associated with hyperoxaluria, the second one is the dihydrate (COD, weddellite)^8–11^, and the final one is the trihydrate (COT, caoxite)^9,12,13^.

Previous investigations have demonstrated that Furosemide (Fm) and Hydrothiazide (Hz) inhibit the dissolution of COM crystals through a surface-controlled mechanism^14^. This inhibition arises from the adsorption of these molecules onto the reactive sites of the COM surface, thereby blocking dissolution pathways. The inhibitory effect was stronger for Fm than for Hz (Fm > Hz), which can be explained by differences in their molecular structures. Fm appears to establish more stable interactions with the crystal surface—such as hydrogen bonding or electrostatic forces—whereas Hz exhibits weaker binding capacity^14^.

The role of citrate and Na^+^ ions on the dissolution of CaOx crystals has been widely studied^15,16^. Citrate is recognized as one of the most effective natural inhibitors of CaOx crystallization. It interacts with surface calcium ions of CaOx crystals, leading to the formation of soluble calcium–citrate complexes. Through this mechanism, citrate reduces crystal aggregation and promotes dissolution, with the extent of this effect depending on both concentration and incubation time. Additionally by theoretical studies, sodium ions in citrate salts may further influence the dissolution process by adsorbing onto the crystal surface or near defect sites, generating local lattice strain that enhances surface deformation and solubility^17^.

Organic modulators, including carboxylic acids, amino acids, and polyphenolic compounds, have been shown to enhance the dissolution of COM crystals^14,18–20^.

Among these, citric and malic acids exert the strongest influence, particularly when applied at concentrations close to 100 µM. Their action is thought to involve coordination with calcium ions, which increases solubility while stabilizing the COM phase. In addition, these molecules are capable of extending the induction period, thereby postponing the onset of nucleation and slowing subsequent crystal growth^21^.

In the present study, we aimed to elucidate the adsorption mechanism and process through both theoretical analysis and characterization techniques, including Fourier Transform Infrared (FTIR), Scanning Electron Microscope (SEM), Energy Dispersive X-ray Spectroscopy (EDX), and X-ray Diffraction (XRD).

This work forms part of our ongoing research project, as referenced in^14,22–24^.

## Experimental

### Materials and methods

Calcium chloride (CaCl_2_) and sodium oxalate (Na_2_C_2_O_4_) (purchased from Sigma-Aldrich) were used to prepare seed crystals. Hydrochloric acid (HCl) and sodium hydroxide (NaOH), were obtained from MERCK, Germany, were used to adjust pH. The coffee plant drink was used as a source of extracted compounds, respectively. Sodium chloride (NaCl) (purchased from Fluka) was of chemical reagent-grade quality. De-ionized water of high quality (conductivity < 0.1 µS cm^− 1^) was utilized for preparing all solutions. The solutions were filtered through a 0.22 μm Millipore filter paper, which had been rinsed beforehand in order to remove traces of wetting agents.

Seed crystals of COM were prepared by dropwise mixing of equimolar aqueous solutions of Na₂C₂O₄ and CaCl₂ at 298 K with a controlled flow (~ 300 mL h^− 1^)^22,25–27^. The mixture was stirred for seven days, stored at ambient temperature for approximately four weeks^25,26^, and the obtained precipitate was separated through filtration, washed thoroughly, and dried at 40 °C^22,28^. The COM phase was verified by analytical methods^26,29^. The Brunauer–Emmett–Teller (BET) nitrogen adsorption analysis, using a Quantachrome TouchWin™ automated gas-sorption analyzer (USA), measured the surface area and porosity of the materials through nitrogen gas adsorption at 77 K, revealing a specific surface area of 3.73 m²/g. Before analysis, the samples were degassed at 200 °C for 2 h.

### Extraction of compounds

To obtain the crude extract, the plant material was repeatedly macerated with ethanol (Al-Brouj, Giza, Egypt). The combined filtrates were filtered and concentrated under reduced pressure at 45 °C, yielding 23 g of a dark brown residue. This material was subsequently defatted with n-hexane, which was supplied by Al-Brouj, Giza, Egypt, re-volatilized, and preserved at 4 °C until further analysis^30,31^.

### Liquid chromatography–linear trap quadrupole–tandem mass spectrometry (LC–LTQ–MS–MS) analysis of extract

LC–MS–MS analysis of the coffee extract (Cf) was carried out using a Shimadzu LC-10 HPLC system interfaced with an LTQ Linear Ion Trap mass spectrometer (Thermo Finnigan, San Jose, CA). Separation was performed on a Grace Vydac Everest Narrowbore C-18 column (100 mm × 2.1 mm i.d., 5 μm, 300 Å). A 2 µL aliquot was injected via an autosampler, and ion signals were monitored over an m/z range of 100–2000. The chromatographic program was 35 min in total: an initial 5 min isocratic elution with 5% acetonitrile (AcN) containing 0.05% formic acid (FA), followed by a 25 min linear gradient to 95% AcN with 0.05% FA, and a final 5 min re-equilibration at the starting conditions. Raw datasets were processed using Xcalibur (version 3.1.6610) and converted to mzXML format with MSConvert (ProteoWizard suite)^32^. Molecular networking was constructed through the Global Natural Products Social Molecular Networking (GNPS) platform, and the obtained spectra were matched against GNPS reference libraries and publicly available datasets.

### Nuclear magnetic resonance (NMR) analysis

A Jeol EX-400 spectrometer (400 MHz) was used to acquire ¹H NMR spectra. Heteronuclear Single Quantum Coherence (HSQC) spectra were registered at 298 ºK on a Bruker Avance 600 MHz instrument, incorporating a TCI CRPHe TR-¹H probe and a 5 mm-EZ CryoProbe, suitable for ¹³C, ¹⁵N, and ¹⁹F detection. Chemical shifts were calibrated against the residual signals of DMSO-d₆ at δH (2.50 ppm), δC (39.52 ppm), respectively^33–35^.

Using 718 titrino, Metrohm as an automatic titrator, which has a 200 ml cell (double-walled) to perform dissolution under an atmosphere of nitrogen at 37.0 ± 0.1 °C by circulating thermostatically^25,26,36^. Filling the cell with a definite volume of solutions, keeping pH 6.0 ± 0.1 as follows: de-ionized water, NaCl, CaCl_2_, Na_2_C_2_O_4_, and the Cf extract solutions, and finally a pre-weighed amount of COM seed crystals to start the dissolution experiments^14,22,23^. A constant electromotive force (emf) maintained for at least 30 min was used to verify the stability of the relative degree of undersaturated solution. The change in emf values with time (every one minute), recorded by an automatic titrino device using the constant-composition method, was used to calculate the reaction rate.

Density functional theory (DFT), performed in Gaussian 16, was applied to study the structural optimization properties of The Highest Occupied Molecular Orbital–Lowest Unoccupied Molecular Orbital (HOMO-LUMO) energy levels of the assessed structure^37–39^. The hybrid Becke, three-parameter, Lee–Yang–Parr (B3LYP) functional was employed to ensure accurate predictions^40^, which is widely recognized for its accuracy in similar materials. This function provides a balance between computational efficiency and precision in predicting structural, electronic, and chemical properties, making it well-suited for our system. The 6-311G(d) basis set was chosen for its effective trade-off between accuracy and computational cost^41–44^, as it is commonly used in comparable studies^45–48^. To verify the stability of the optimized structures, vibrational frequency calculations were performed on the minimum energy geometries. The absence of imaginary frequencies confirmed that the optimized structures correspond to a true minimum on the potential energy surface. Additionally, the density of states (DOS) was analyzed using Multiwfn software^49^ to gain deeper insights into the electronic structure.

The solid obtained at the end of the experiments were isolated by filtration, and characterized as COM seed crystals with/without the additive by FTIR, SEM, EDX, and XRD.

FTIR spectroscopy of the prepared samples was recorded using a PerkinElmer FTIR spectrometer (USA) in the range of 4000–400 cm^−^¹. SEM was acquired using a JEOL SEM equipped with an EDX detector (JEOL, Japan), operated at an accelerating voltage of 80 kV. XRD patterns were recorded using a Bruker D8 Advance diffractometer (Bruker, Germany) equipped with a Cu–Kα radiation source (λ = 1.5406 Å) operated at 40 kV and 40 mA, with a scanning rate of 0.2° min^−^¹.

## Results and discussion

**Fig. 1:**
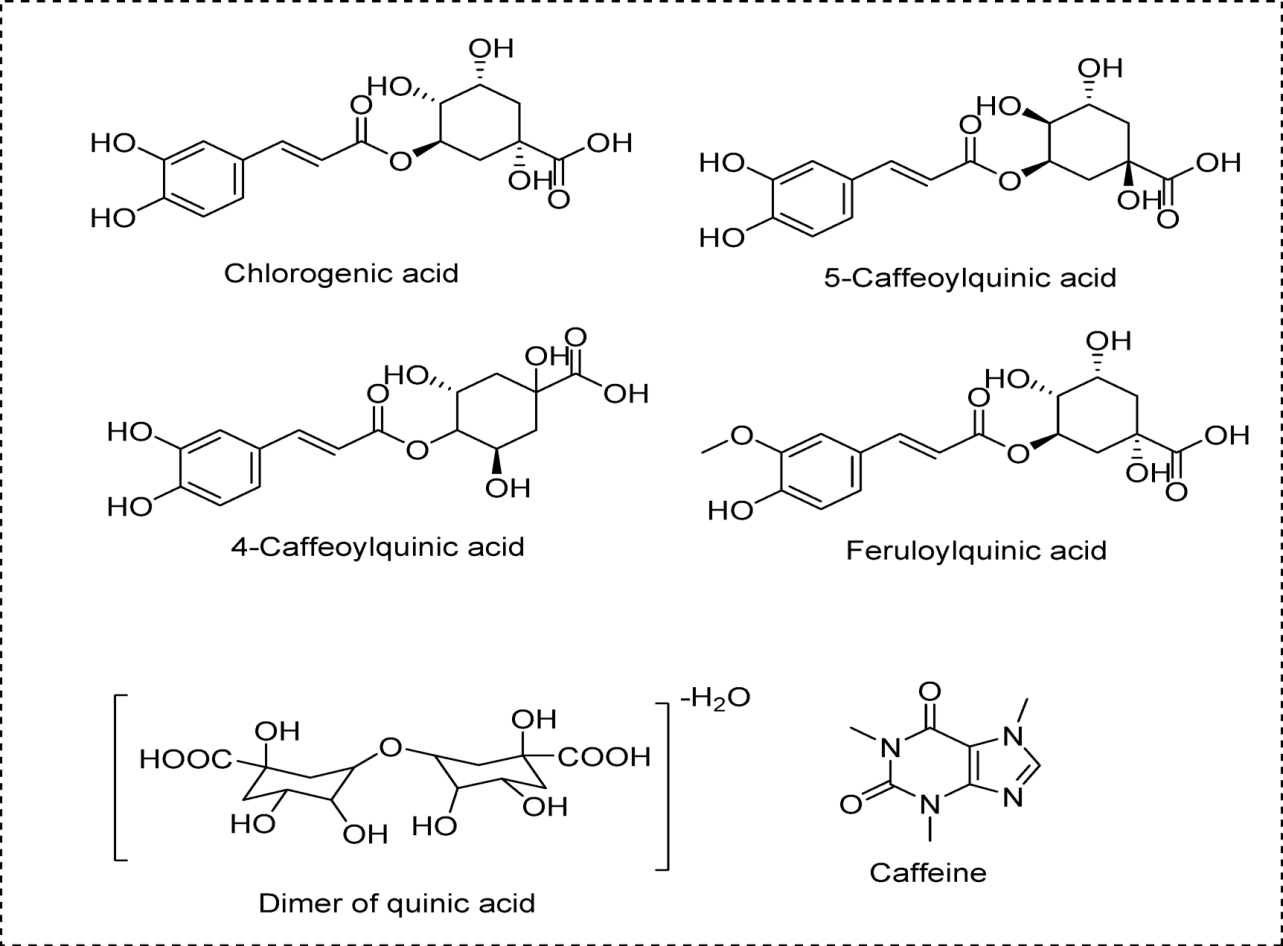
Chemical structures of some major metabolites identified from Cf extract additive.

In the Cf extract additive using LC–LTQ–MS–MS analysis, some of the major components were identified (as shown in Table 1 and additional data supporting the findings of this study are provided in the Supplementary Materials, Fig.S1(a-c) and Fig. S2(a, b)). All compounds were detected and identified as quinic acids. Chlorogenic acid, 5-caffeoylquinic acid, and 4-Caffeoylquinic acid, with the chemical formula C_16_H_18_O_9_, appeared at m/z 353.0875, feruloylquinic acid, with the molecular formula C_17_H_20_O_9_, was shown at m/z 369.2957, and a dimer of quinic acid, with the chemical formula C_14_H_22_O_11_-H_2_O, appeared at m/z 365.1075. The chemical structures of the detected metabolites are shown in Fig. 1.

**Table 1:** Identified metabolites of Cf extract additive analyzed by LC-MS/MS and compared with literature data.

t_*R*_ (min)	Identified compound/chemical class	Formula	[M-H]^+a^/[M-H]^−b^ (m/z)	References
1.467	Chlorogenic acid/quinic acids	C_16_H_18_O_9_	353.0875	^50^
1.467	5-Caffeoylquinic acid/quinic acids	C_16_H_18_O_9_	353.0875	^50^
1.467	4-Caffeoylquinic acid/quinic acids	C_16_H_18_O_9_	353.0875	^51^
6.634	Feruloylquinic acid/quinic acids	C_17_H_20_O_9_	369.2957	^52^
0.374	Dimer of quinic acid/quinic acids	C_14_H_22_O_11_-H_2_O	365.1075	^53^

^a^ negative ionization mode.

^b^ positive ionization mode.

### One-dimensional (1D) and two-dimensional (2D) NMR

**Fig. 2:**
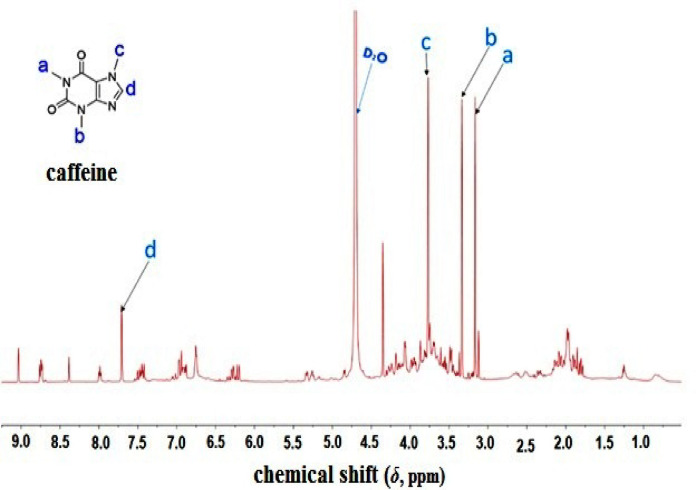
^1^H-NMR of Cf extract showing caffeine peaks as main components.

**Fig. 3:**
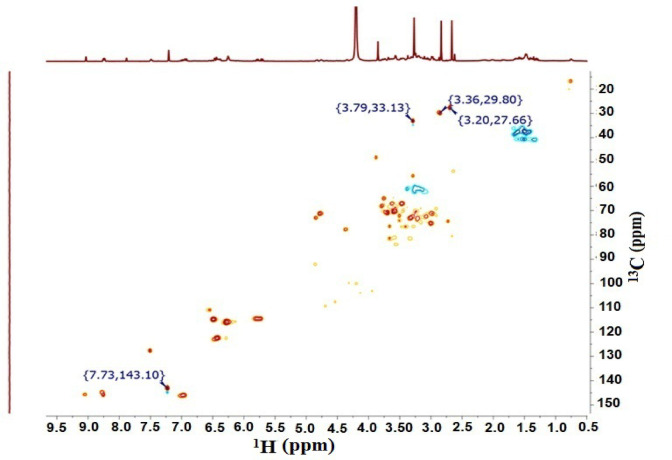
2D ^1^H-^13^ C HSQC NMR of Cf extract showing ^1^H-^13^ C correlations of the main component caffeine.

1D and 2D NMR of coffee extract showed caffeine as a major component, as indicated by the highest intensity peaks in both 1D and 2D NMR analyses. Table 2 illustrates the ^1^H and ^13^C shifts of caffeine in coffee extract. Figs. 2 and 3 illustrate ^1^H shifts and ^1^H-^13^C correlations of caffeine in ^1^H-NMR and HSQC, respectively.

**Table 2:** ^1^H and ^13^C shifts of caffeine.

No.	^1^H shifts (ppm)	^13^C shifts (ppm)
A	3.20	27.66
B	3.36	29.80
C	3.79	33.13
D	7.73	143.10

The ionic concentrations present in the solution were determined through mass balance considerations together with electroneutrality conditions, following the procedure outlined earlier^23,24^. These calculations employed the appropriate thermodynamic equilibrium constants (*K*) for the formation of the different ionic associates. The activity coefficients of the species were evaluated using the extended Debye–Hückel relationship as modified by Davies^54^.

For sparingly soluble salts of the type M_a_A_b_, the dissolution rate (*R*) normalized to the area of the crystal surfaces, at time (*t*), can be represented by Eq. (1):

1 $$\:R\hspace{0.17em}=\hspace{0.17em}d\:\left[M_{a}A_{b}\right]\:/\:dt\hspace{0.17em}=\hspace{0.17em}k\:s\:\:\sigma^{n}$$

Where, *k* is the intrinsic dissolution rate constant determined from the intercept of typical plot of −1og *R* against -log *σ*, *s* is proportional to the number of dissolution sites available on the seed crystals, and is set to 1 (normalized), *n* is the apparent reaction order determined from the slope of linear plot, and σ denotes the relative degree of undersaturation^23,24,55^.

**Fig. 4:**
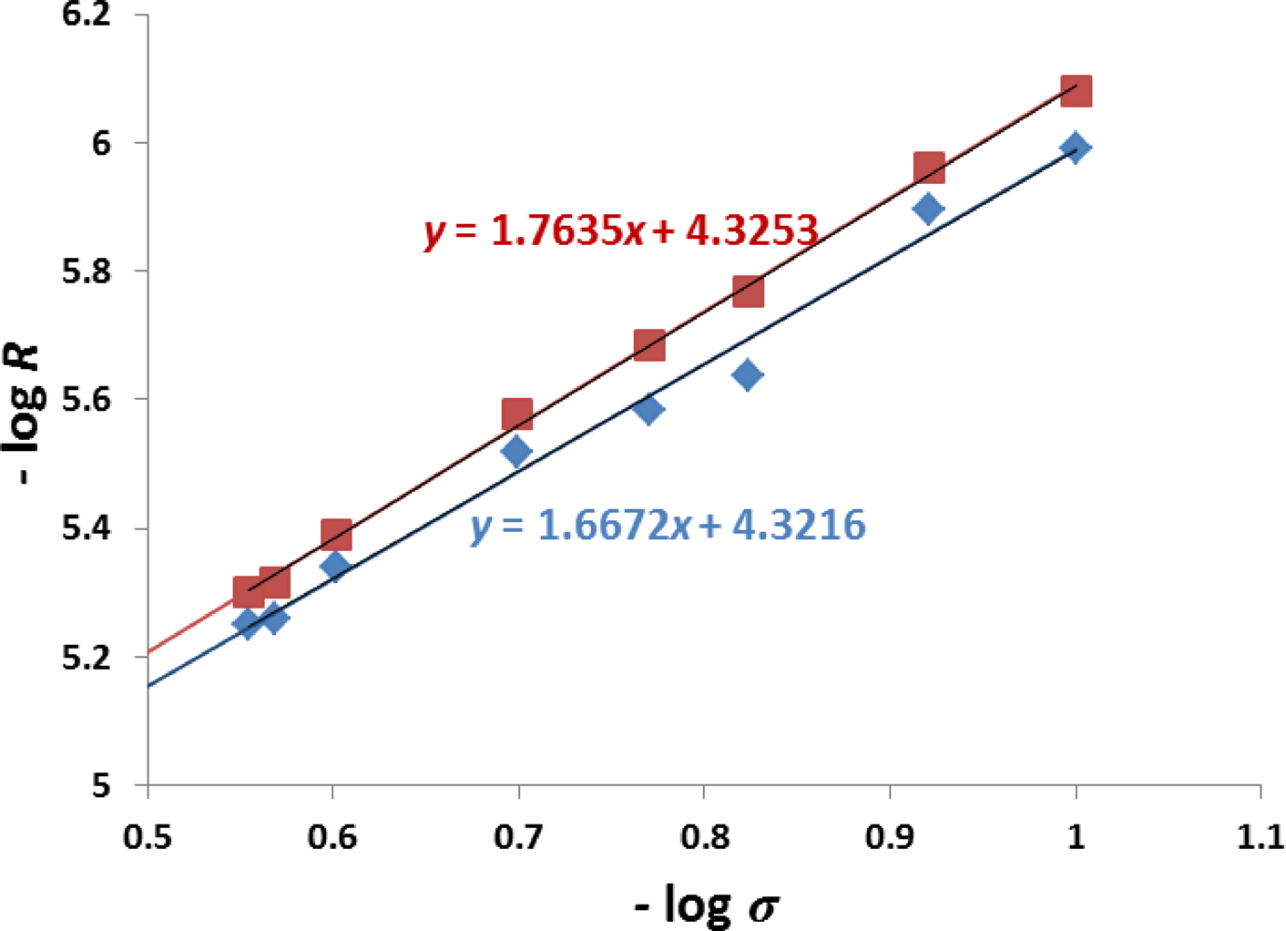
Plots of – log *R* against –log *σ* (with 1 × 10^− 7^ M Cf extract ($\color{red} {\blacksquare}$), without Cf extract as additive ($\color{blue} {\blacklozenge}$) for dissolution of COM seed crystals at 37.0 ± 0.1 °C, *I* = 0.15 mol dm^− 3^, pH = 6.0 ± 0.1, and 10.0 mg of seed.

From Fig. 4, the rate constants of dissolution reaction in the absence and the presence of inhibitor were 4.769 × 10^− 5^ mol^− 1^ m min^− 1^, and 4.728 × 10^− 5^ mol^− 1^ m min^− 1^, respectively.

The relative degree of undersaturation (*σ*) is defined in Eq. (2)^55^:

2 $$\sigma\:=\:(H_{0}^{1/2}-H^{1/2})\:/\:H_0^{1/2}$$

In which *H*^1/2^ is the molar concentration product of COM, ([Ca^2+^] [C_2_O_4_^2−^]) in the solutions, depending on the value of *σ*, and *H*ₒ^1/2^ (the solubility value at the same ionic strength), was equal 1.994 × 10^− 4^ mol dm^− 3^.

The prepared COM seed crystals without and with Cf extract inhibitor elucidated using FTIR, SEM, XRD, and EDX. Using the constant-composition method, the rate at which the seed crystals dissolved was investigated. Dissolution experimental conditions for the Cf extract inhibitor are summarized in Table 3, where *T*_Ca_ and *T*_C2O4_ represent the total molar concentrations of calcium and oxalate (*H*^1*/*2^), respectively. From the results, at *t* = 37.0 ± 0.1 °C, pH = 6.0 ± 0.1, and ionic strength, *I* = 0.15 mol dm^− 3^, the rates of dissolution of COM seed crystals are calculated. From the Table 3, it was found that the rates increased with increasing the relative *σ*.

**Table 3:** Dissolution of COM synthetic crystals in the absence and the presence of Cf extract additive, *T*_Ca_: *T*_C2O4_ = 1:1, at *t* = 37.0 ± 0.1 °C, pH = 6.0 ± 0.1, and *I* = 0.15 mol dm^− 3^.

T_Ca_ x10^− 4^ (mol dm^− 3^)	σ x 10^− 2^	Seed (mg)	Stirring Speed (rpm)	Rate x10^− 6^ (mol m^− 1^ min^− 1^) (COM seed crystals)	Rate x10^− 6^ (mol m^− 1^ min^− 1^) (in the presence of Cf extract additive)
1.791	10	10	300	1.023	0.832
1.751	12	10	300	1.271	1.095
1.692	15	10	300	2.301	1.712
1.652	17	10	300	2.606	2.071
1.592 1.493	20 25	10 10	300 300	3.027 4.581	2.652 3.985
1.453	27	10	300	5.520	4.814
1.433	28	10	300	5.955	4.999

Plotting of –log *R* against *–*log *σ* in the absence and presence of inhibitor Cf extract is illustrated in Fig. 4. From the figure, both the orders of dissolution reaction of COM crystals in the absence and the presence of Cf inhibitor were calculated from the slopes of the straight lines and found to be equal 2, which suggest that the dissolution reactions may follow a film surface control mechanism^56^.

Table 4 illustrates the dissolution rates of COM seed crystals inhibited in the presence of different concentrations of Cf extract inhibitor. Moreover, a significant decrease in the rates of dissolution of COM seed crystals, with the gradual addition of this extract, was detected. From Table 4, a concentration of Cf extract inhibitor as low as 10.000 × 10^− 6^ mol dm^− 3^_,_ reduced the dissolution rate by at least 18.519%, compared to that in the absence of an additive at the same relative degree of undersaturation (*σ* = 0.2). The active dissolution sites on the seed crystal COM surface, may be blocked as the concentration of additive molecules increased through adsorption on these active sites, decreased their numbers and decreased the dissolution rates of COM seed crystals.

**Table 4:** Dissolution of COM seed crystals in the presence of different concentrations of the Cf extract inhibitor, *T*_Ca_: *T*_C2O4_ = 1:1 at 37.0 ± 0.1 °C, *σ* = 20.0 × 10^− 2^, *I* = 0.15 mol dm^− 3^, pH = 6.0 ± 0.1, and 10.0 mg of seed.

[Inhibitor] 10^− 6^ mol dm^− 3^	Rate, 10^− 6^ mol m^− 1^ min^− 1^	% Inhibition
-	3.027	-
2.000	2.895	4.367
2.220	2.879	4.854
2.500	2.863	5.406
3.000	2.828	6.579
4.000	2.786	7.956
5.000	2.712	10.417
6.000	2.607	13.859
7.000	2.601	14.084
8.000	2.554	15.625
9.000	2.486	17.857
10.000	2.466	18.519

Following the approach originally described by Nancollas and Zawacki^24,25,57^, the kinetic Langmuir-type isotherm equation was applied to describe the adsorption process, as expressed in Eq. (3):

3 $$\theta\:=\:R_0\:/\:(R_0-R_i)\hspace{0.17em}=\hspace{0.17em}1+1/\:K_LC\:$$

**Fig. 5:**
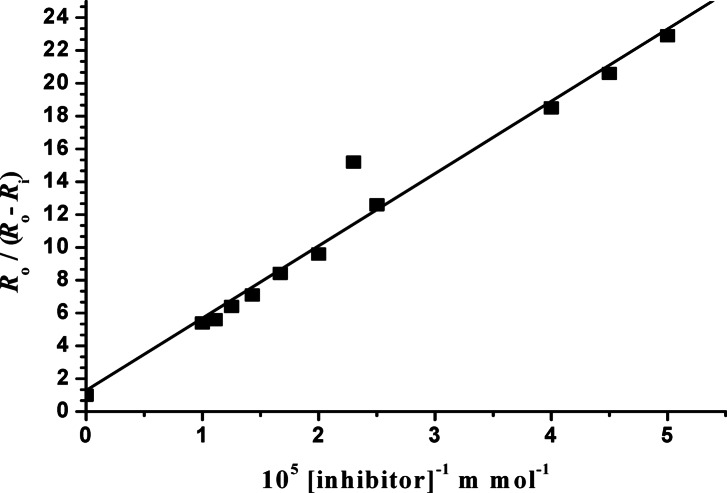
Plot of *R*ₒ/(*R*ₒ - *R*_*i*_) against [inhibitor]^−1^ at 37.0 ± 0.1 °C, *σ* = 20.0 × 10^− 2^, *I* = 0.15 mol dm^− 3^, pH = 6.0 ± 0.1, and 10.0 mg of COM seed crystals.

Where $\theta$ is the surface coverage, *R*ₒ is the growth rate in the absence of additives, *R*_i_ is the actual growth rate in the presence of the inhibitor, *C* is the fraction of moles contributed by the impurity in the solution, which represents the concentration of inhibitor, and *K*_L_ is the adsorption constant (affinity constant). The applicability of the Langmuir model for the present system was confirmed by the linearity of the experimental plots Fig. 5^24,25,57^. The values of each variable in Eq. (3) are summarized in Table 5.

Typical plot according to the last Eq. (3), illustrated in Fig. 5. The suitability of this simple adsorption isotherm at *σ* = 0.2, and the stirring rate did not affect the reaction rates, supported the surface mechanism. The value of *K*_L_ was calculated from the slope of the line and was found to be 2.274 × 10^4^ dm^3^ mol^− 1^. This value reflects the high adsorption affinity at a low degree of relative undersaturation in the presence of Cf extract compounds.

The rate of dissolution in the presence of an additive (*R*_i_) is given by Eq. (4), depending on the surface coverage^58^.

4 $$\:R_{i}\:=\:R_\theta\:(1\:-\theta)$$

The values of $\theta$ in the presence of an additive are reviewed in Table 5.

**Table 5:** Values of surface coverage in case of the presence of Cf extract inhibitor at *σ* = 0.15, *t* = 37.0 ± 0.1 °C, *I* = 0.15 mol dm^− 3^, and pH = 6.0 ± 0.1 using emf.

[inhibitor], 10^− 6^ mol dm^− 3^	[inhibitor]^−1^, 10^5^ dm^3^ mol^− 1^	*R*_i_, 10^− 6^ mol m^− 1^ min^− 1^	Rₒ/(Rₒ - *R*_i_)	ɵ, 10^− 1^	C_i_/ɵ, 10^− 5^
-	-	3.027	1.000	-	-
2.000	5.000	2.895	22.899	0.437	4.579
2.220	4.500	2.879	20.599	0.485	4.577
2.500	4.000	2.863	18.499	0.541	4.625
3.000	2.300	2.828	15.199	0.658	4.559
4.000	2.500	2.786	12.568	0.796	5.028
5.000	2.000	2.712	9.600	1.042	4.799
6.000	1.670	2.607	7.216	1.386	4.329
7.000	1.429	2.601	7.100	1.408	4.972
8.000	1.250	2.554	6.400	1.563	5.122
9.000	1.110	2.486	5.599	1.786	5.039
10.000	1.000	2.466	5.399	1.852	5.399

**Fig. 6:**
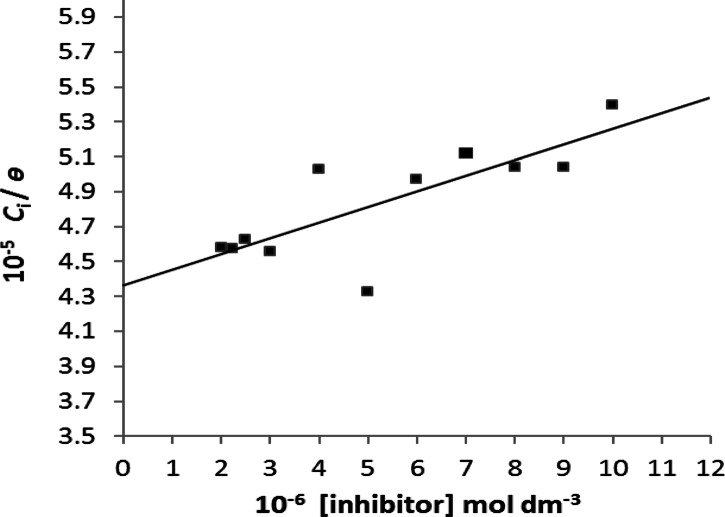
Plots of *C*_i_/$\theta$ against [additive] at 37.0 ± 0.1 °C, *σ* = 20.0 × 10^− 2^, *I* = 0.15 mol dm^− 3^, pH = 6.0 ± 0.1, and 10.0 mg of COM seed crystals.

Fig. 6, illustrates the plotting of *C*_i_/$\theta$ against concentration of inhibitor (*C*_i_), a straight line was obtained with an intercept equal to 1/*K*_ads_ by applying the Langmuir isotherm Eq. (5)^58^. Where *K*_ads_ is the adsorption equilibrium constant.

5 $$\:C_i\:/\:\theta\:=\:C_i\:+\:1\:/\:K_{ads}$$

From Fig. 6, the value of *K*_ads_ was determined from the intercept of the straight line. It was found to be equal to 2.291 × 10^4^ dm^3^ mol^− 1^.The Gibbs free energy of adsorption (Δ*G*_ads_), representing the free energy change associated with transferring the system from an undersaturated solution to equilibrium in the presence of the inhibitor, was calculated Using the Eq. (6) and It is found to be equal − 36.232 KJ/mol

6 $$\:G_{ads}\hspace{0.17em}=\hspace{0.17em}-\hspace{0.17em}R\:T\:lnK_{ads}$$

Where *R* is the universal gas constant (8.314 J mol^− 1^ K^− 1^) and T is the absolute temperature at which the adsorption measurements were performed (310 K). From the values of *K*_ads_ and Δ*G*_ads_, it was found that there is strong adsorption of the Cf extract inhibitor on the surface of COM seed crystals^58–60^.

### DFT study

**Fig. 7:**
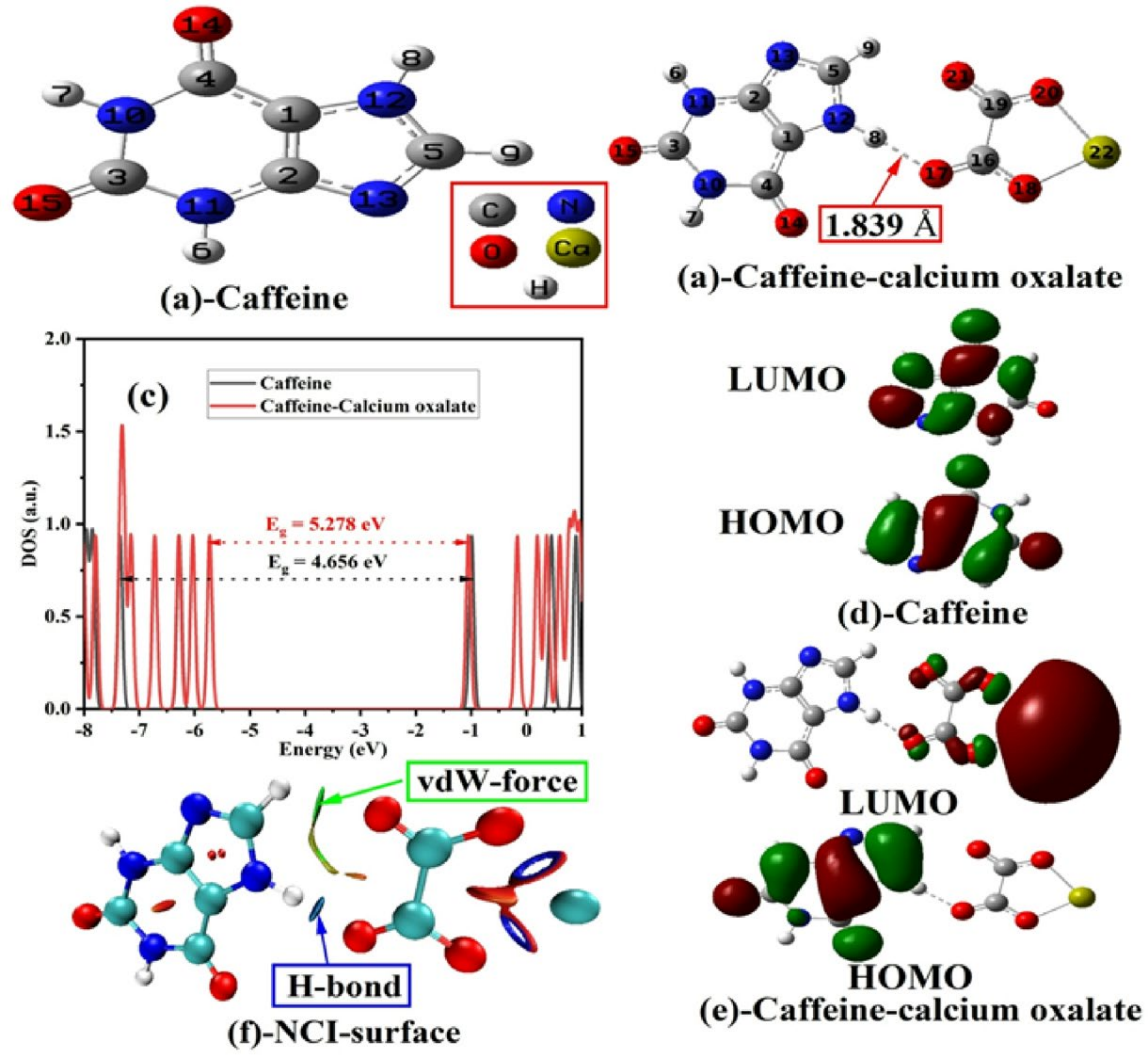
Structural and electronic interaction analysis between caffeine and calcium oxalate. (**a**) Optimized molecular structure of caffeine. (**b**) Optimized structure of the caffeine–calcium oxalate complex, highlighting the H-bond distance (1.839 Å) between caffeine and the oxalate moiety. (**c**) Density of states (DOS) comparison showing the bandgap reduction from 5.278 eV (caffeine–calcium oxalate) to 4.656 eV (caffeine), indicating electronic interaction. (**d**) Frontier molecular orbitals (HOMO and LUMO) of isolated caffeine. (**e**) HOMO and LUMO of the caffeine–calcium oxalate complex showing orbital redistribution due to interaction. (**f**) Non-covalent interaction (NCI) surface revealing hydrogen bonding and van der Waals (vdW) interactions stabilizing the complex.

The interaction between caffeine and CaOx in an aqueous medium was thoroughly analyzed using structural, electronic, and non-covalent interaction data, as illustrated in Fig. 7; Table 6. Fig. 7a displays the optimized geometry of isolated caffeine, while Fig. 7b shows the structure of the caffeine– CaOx complex. A significant hydrogen bond with a distance of 1.839 Å is observed, indicating a hydrogen bond between caffeine and the oxalate moiety. According to Table 6, the C3–N10 bond slightly shortens from 1.397 to 1.374 Å, while the C3– O15 bond elongates from 1.223 to 1.246 Å, suggesting a redistribution of electron density due to the interaction. The formation of a new N12-H8…O12 hydrogen bond, with a length of 1.839 Å, further confirms the hydrogen bonding between caffeine’s nitrogen and the oxygen atom of CaOx. Additionally, the N10–C3–N11 and C4–C1–C2–N13 angles undergo slight changes, indicating a mild geometric rearrangement. The density of states (DOS) plots in Fig. 7c reveal an increase in the energy gap (*E*_g_) from 4.656 eV (caffeine) to 5.278 eV (complex), indicating that the interaction leads to a widening of the *E*_g_ and a potential reduction in electronic conductivity. The *E*_g_ was calculated using the Eq. (7)^37,38^:

7 $$\:E_g\:=\:E_{LUMO}-E_{HOMO}$$

This equation represents the difference between the energy levels of LUMO and HOMO. Fig. 7d and e show the HOMO–LUMO isosurfaces for caffeine and the complex, respectively. In the complex, electron density is delocalized across both molecules, indicating orbital hybridization and electronic interaction. Fig. 7f (NCI surface) highlights the non-covalent interactions (NCIs) responsible for complex formation. Blue regions represent hydrogen bonds (H-bonds), while green regions correspond to van der Waals (vdW) forces. These interactions stabilize the caffeine– calcium oxalate complex without the formation of strong covalent bonds.

The adsorption energy (*E*_a_) is calculated to be − 0.273 eV, indicating an exothermic and spontaneous process. The relatively low value of *E*_a_ (typically less than − 0.5 eV) is a hallmark of physisorption, where weak forces (e.g., hydrogen bonding and vdW interactions) govern the interaction, rather than chemisorption. The *E*_a_ was calculated using the Eq. (8):

8 $$\:E_a\:=\:E_{complex}-(E_{caffeine}\:+\:E_{CaOx})$$

Where *E*_complex_ = −44009.820 eV is the total energy of the caffeine–calcium oxalate system, and *E*_caffeine_ = −15305.276 eV and *E*_CaOx_ = −28704.271 eV are the total energies of the individual components. The negative value confirms that complex formation is thermodynamically favourable and occurs via physisorption. In summary, the results from Fig. 7; Table 6 demonstrate that caffeine interacts with calcium oxalate through physisorption, dominated by hydrogen bonding and van der Waals interactions. This interaction leads to observable changes in molecular geometry, a widening in the electronic energy gap, and stabilization of the complex, as indicated by the calculated adsorption energy of − 0.273 eV. These findings are consistent with experimental results, supporting the conclusion that physisorption governs the interaction between caffeine and calcium oxalate. This agreement with experimental data further validates the theoretical model, providing essential insights into the binding behavior of caffeine in systems involving calcium oxalate^61,62^. However, these effects may differ in real or synthetic urine, which has a far more complex composition. Therefore, further investigation in physiologically relevant media is warranted.

**Table 6:** Some important quantum parameters like bond length (Å), dihedral, bond angles (degrees), and adsorption energy (*E*_a_) for caffeine and caffeine-calcium oxalate in water.

Compound	C3-N10	C3-O15	C1-C2	H8….O12	N10-C3-N11	C4-C1-C2-N13	E_a_ (eV)
Caffeine	1.397	1.223	1.383	-	114.531	179.994	-
Caffeine-calcium oxalate	1.374	1.2462	1.382	1.839	116.821	179.995	−0.273

### Characterization of COM seed crystals in the absence and the presence of the Cf extract inhibitor

To describe the nature, habit, elemental composition, and the purity, several analytical techniques are employed.

**Fig. 8:**
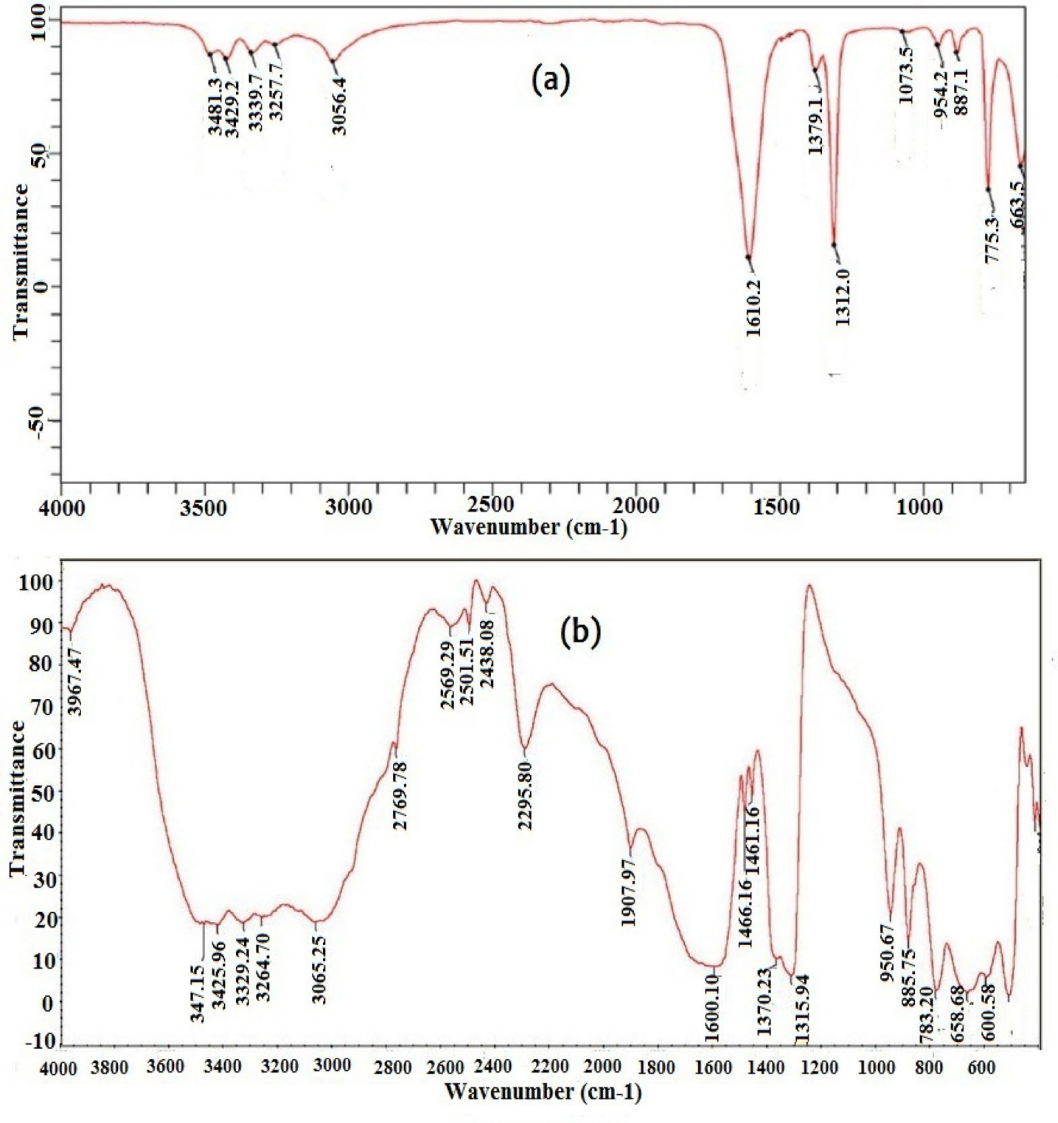
FTIR spectra of (**a**) COM seed crystals and (**b**) COM seed crystals with inhibitor additive.

The FTIR Spectrum of COM seed crystals prepared and after addition of Cf extract is illustrated in Fig. 8. The IR spectrum of Fig. 8a exhibited a characteristic grouping of bands between 3481/cm − 3056/cm that are ascribed to symmetric and asymmetric OH stretch. The strong band at 1610 − 1600/cm and the sharp band at 1379/cm and 1312/cm are assigned to n(C = O) stretch and n(C-O) stretch. The bands at 1073/cm, 887/cm, 775/cm, and 663/cm indicate to (C-C) stretch, (C-H) bending, (O-H) bending, and (O-C-O) bending. It shows the expected COM, with no additional functional groups—again consistent with intact COM structure and absence of organic adsorbates^29,63^. Fig. 8b, illustrates similar features with slight shifts due to interactions within the modified system. The broad band at 3474–3264/cm indicates (O–H) stretching hydrogen-bonding effects. New bands ranging from 2769.78/cm to 2295.80/cm, these subtle spectral changes are typical of surface adsorption or hydrogen-bonding interactions^64,65^between caffeine and oxalate/calcium surface sites; such interactions modify band intensities without creating new crystalline phases.

The peaks at 1600 − 1315/cm correspond to oxalate stretching vibrations, consistent with COM structure. Multiple bands appearing between 950–600/cm further represent bending and deformation modes of the oxalate group. The overall spectra of both samples confirmed the presence of calcium oxalate monohydrate, with minor spectral variations in sample (b) suggesting molecular interactions influencing the vibrational environment^29,65^.

**Fig. 9:**
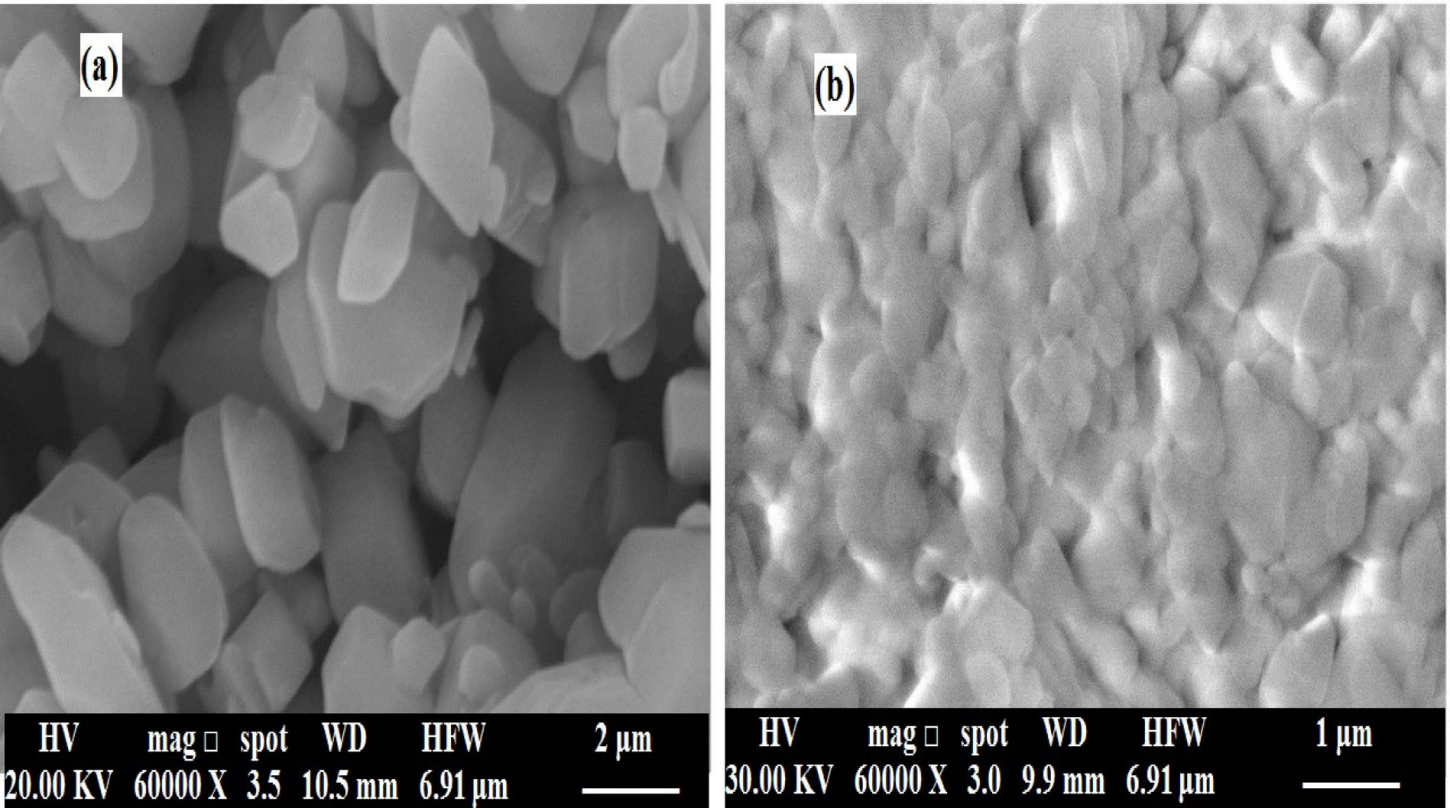
SEM of (**a**) the prepared COM seed crystals and (**b**) after adsorption of Cf extract inhibitor on the COM surface.

Fig. 9 shows an SEM image of the crystal at low magnification. Fig. 9a, exhibits the well-defined prismatic and elongated crystals with sharp faceted edges and smooth surfaces that refer to the COM crystals prepared^64,65^. The surfaces appear clean and homogeneous, indicating undisturbed crystal growth in the absence of additives. This description is characteristic of stable COM seed crystals formed under controlled conditions. Fig. 9b reveals that the crystals appear less defined, aggregated, and partially distorted, with smoother, less faceted surfaces. This loss of sharp crystal boundaries and the appearance of surface roughness suggest adsorption of coffee-derived molecules (such as caffeine or quinic acid derivatives) onto COM surfaces. Such adsorption interferes with normal crystal growth and modifies the surface energy, leading to surface passivation and changes in crystal habit.

**Fig. 10:**
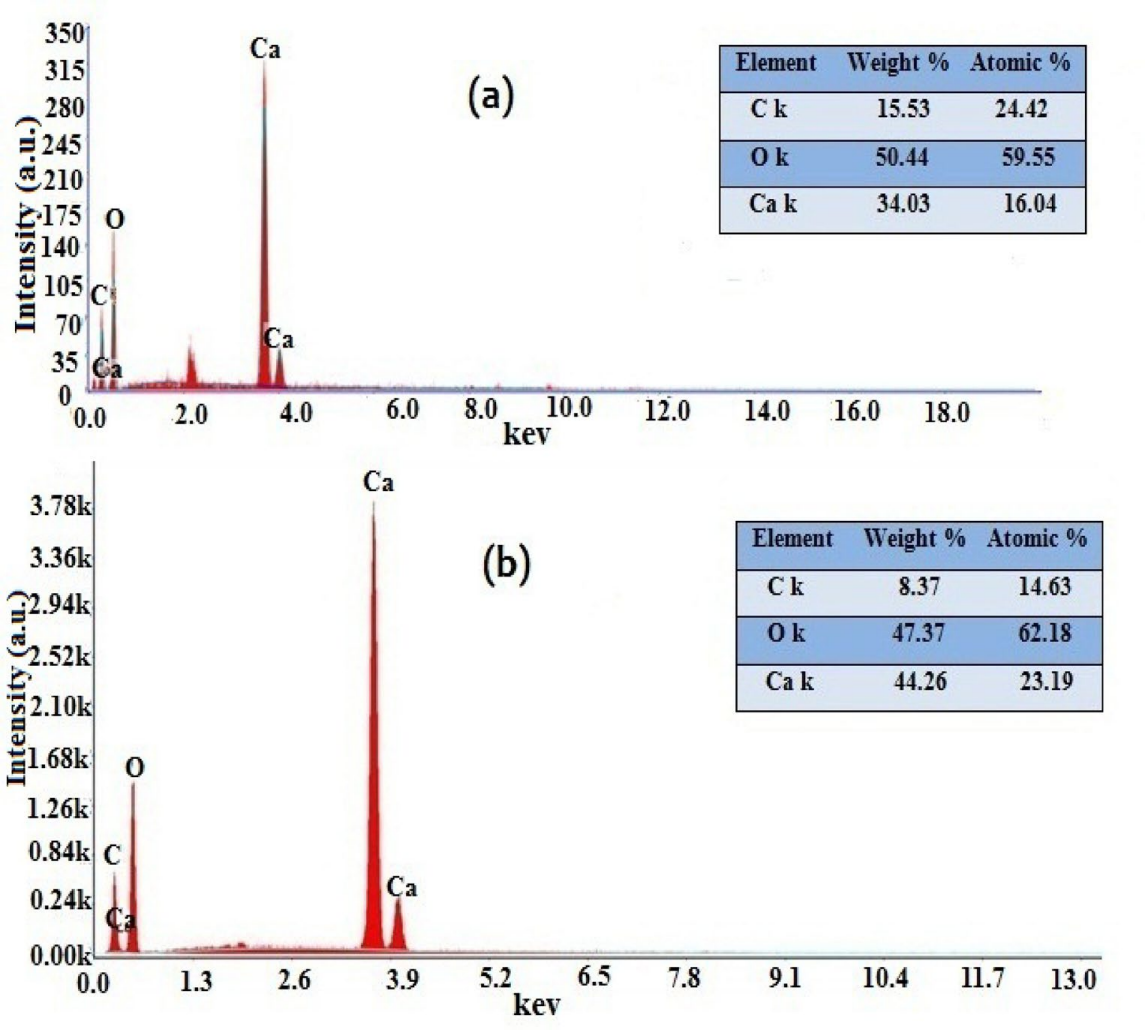
EDX of (**a**) COM seed crystals and (**b**) COM seed crystals in presence of Cf extract inhibitor.

EDX analysis was employed for verification of the elemental composition^66^of the COM seed crystals in the presence and absence of the Cf extract additive, revealing distinct peaks corresponding to the presence of several elements such as calcium, carbon, and oxygen, as shown in Fig. 10. Fig. 10a represents EDX of COM seed crystals in the absence of an inhibitor; the elements percent of Ca, C, and O are 34.03%, 15.53% and 50.44% and the number of atom ratios 0.851, 1.290, and 3.152 can be calculated. While the percentage of the same elements in order are 44.26%, 8.37%, and 47.37% and the number of atom ratios 1.107, 0.698, and 2.961, for COM crystals in the presence of Cf extract inhibitor, shown in Fig. 10b.

EDX analysis revealed an increase in surface Ca, accompanied by decreases in oxygen and carbon. This indicates a true surface chemical change, where oxalate/organic carbon is removed, potentially through oxidation to CO_2_ or desorption, resulting in a relatively Ca-enriched surface. Dissolution of COM crystals releases Ca^2+^ and C_2_O_4_^2−^ into the solution. If oxalate is partially oxidized to CO_2_ or lost as a gas, the measured surface carbon decreases, while Ca in solution may subsequently re-adsorb or precipitate as Ca-rich surface phases, leading to an overall increase in surface Calcium^66^. The desorption of organic carbon from coffee-derived compounds further reduces the surface C signal and exposes the underlying Ca-rich crystal faces. Cf extract strongly inhibited COM seed crystals, which inhibits nucleation, crystal growth, and aggregation, thereby reducing the tendency of released Ca^2+^ ions to bind back to oxalate or the crystal surface. In COM crystals, surface carbon is mainly derived from oxalate (C_2_O_4_^2−^). Coffee polyphenols interact with hydrogen and may partially oxidize oxalate; however, in this study, all interactions are physical, which limits oxalate release into the solution. Adsorption of coffee constituents onto the crystal surface further decreases oxalate availability, thereby contributing to the observed effects^65,66^.

The higher Ca content in Fig. 10b suggests enhanced crystallinity or a higher degree of crystal packing, which is consistent with the stronger Ca peaks observed in the spectrum. The relative decrease in carbon content may indicate surface modification effects^65^.

**Fig. 11:**
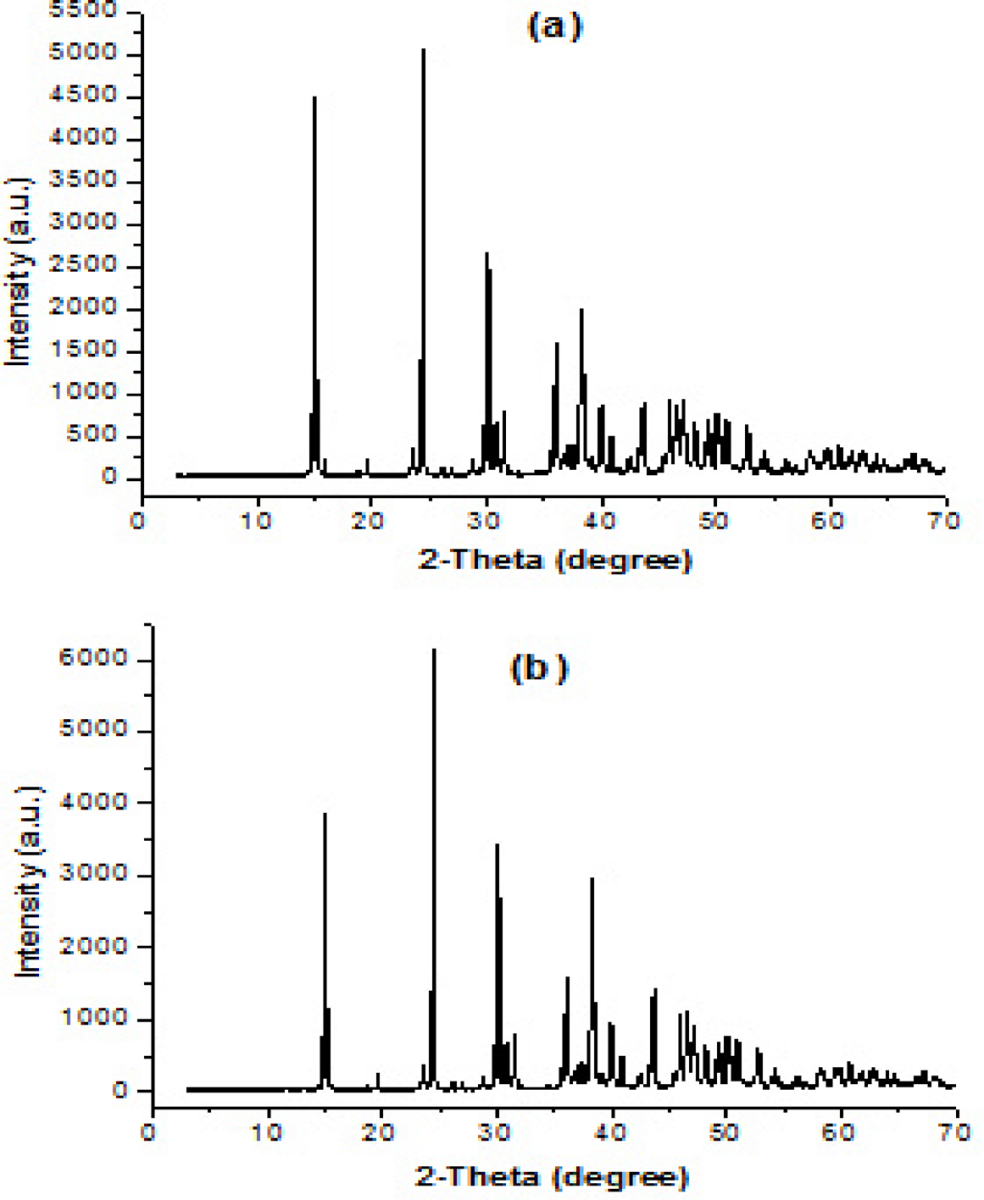
XRD of (**a**) COM seed crystals, (**b**) COM seed crystals in presence of Cf inhibitor.

XRD of the control sample Fig. 11(a, b) shows diffraction peaks indexed to whewellite (COM crystals), with the most intense reflections corresponding to the (101), (121) and (202) planes, consistent with reference data for COM (ICDD, International Centre for Diffraction Data) PDF card No. 20–0231^63–66^. Fig. 11a, displays sharp and intense diffraction peaks at approximately 2$\theta$ ≈ 14.9°, 24.4°, 30.1°, 34.3°, and 38.1°, which are characteristic of monoclinic COM. The sharpness and high intensity of these reflections indicate a highly crystalline structure with well-ordered lattice arrangement^67^. The XRD pattern of the sample treated with Cf extract (Fig. 11b) exhibits the same monoclinic COM phase; however, a slight decrease in peak intensity is observed. This reduction suggests a decrease in crystallite size and possible lattice distortion. The average crystallite size was calculated to be 0.420 nm for the untreated COM and 0.417 nm for the sample treated with the inhibitor, supporting the hypothesis that surface adsorption of extract constituents blocks active growth sites and suppresses long-range lattice ordering^64,65^.

The combined XRD, FTIR, SEM, EDX, and computational findings demonstrated that Cf extract (caffeine) inhibits the dissolution of COM via a surface-adsorption mechanism. XRD confirms that the COM lattice remains unchanged, while FTIR shows no new vibrational bands, excluding chemical bonding or lattice incorporation. SEM reveals altered crystal habit and increased surface roughness in the presence of Cf (caffeine), and EDX detects slight shifts in surface elemental ratios, both indicating surface modification rather than bulk substitution. From the present work, the experimental results align with the computational observation, which illustrated that caffeine binds to surface of COM through hydrogen bonding supported by van der Waals interactions. Together, these results establish that COM dissolution inhibition is governed by physisorption, which selectively modifies surface growth without affecting the internal crystal structure.

## Conclusion

In this study, the dissolution behavior of COM seed crystals, the most thermodynamically stable phase of calcium oxalate, was examined in the absence and presence of an aqueous coffee extract. Under the applied experimental conditions, COM dissolution crystals followed a second-order reaction and were independent of fluid dynamics. The low activation energy (*E*_a_) further indicated that the dissolution process is governed by a surface-controlled mechanism. A concentration as low as 10 × 10^− 6^ mol dm^− 3^ decreased the dissolution rate of COM seed crystals by 18.519%.

The applicability of the Langmuir isotherm and the obtained *K*_L_ reflected a high adsorption affinity at low undersaturation levels in the presence of the additive. The values of *K*_ads_ and Δ*G*_ads_ further supported a surface-adsorption mechanism. DFT analysis indicated the formation of hydrogen bonds between caffeine and the oxalate moiety, as well as additional H-bonding interactions involving the nitrogen and oxygen atoms of COM. The adsorption energy (*E*_a_) confirmed a spontaneous, isothermal adsorption process.

Overall, this study demonstrates that coffee-derived metabolites, particularly caffeine and quinic acid derivatives, can physically adsorb onto calcium oxalate monohydrate surfaces, reducing their dissolution through hydrogen bonding and van der Waals interactions. The combined experimental and computational evidence highlights coffee extract as a natural and promising modulator against COM crystals formation.

## Supplementary Information

Below is the link to the electronic supplementary material.

Supplementary Material 1

## Data Availability

All data generated or analyzed during this study are included in this published article.

## References

[1] Kassaw, A. B., Belete, M., Assefa, E. M. & Tarek, A. A. Prevalence and clinical patterns of urolithiasis in sub-saharan africa: a systematic review and meta-analysis of observational studies. *BMC Nephrol.* **25**, 334. 10.1186/s12882-024-03780-y (2024). 39379862 10.1186/s12882-024-03780-y PMC11460051

[2] Collaborators, G. B. D. U. The global, regional, and National burden of urolithiasis in 204 countries and territories, 2000–2021: a systematic analysis for the global burden of disease study 2021. *eClinicalMedicine* **78**, 102924. 10.1016/j.eclinm.2024.102924 (2024). 39640943 10.1016/j.eclinm.2024.102924 PMC11618031

[3] Szymanski, J., Chlosta, P. & Przydacz, M. Epidemiology, diagnostics, and therapeutic strategies in urolithiasis: a comprehensive review. *Przegl Lek Jag Med. Rev.* **77** (2). 10.20452/jmr.2025.19999 (2025).

[4] Szmigielska, A., Skrzypczyk, P. & Tomaszewska, M. Epidemiology and types of urolithiasis. *Pediatr. Med. Rodz.* **15**(1), 22–25. 10.15557/PiMR.2019.0004 (2019).

[5] Daudon, M. & Knebelmann, B. Épidémiologie de La lithiase urinaire. *EMC Urol.* **61**, 1–27. 10.1016/S1762-0953(18)60342-5 (2018).

[6] Peerapen, P. & Thongboonkerd, V. Kidney Stone Prevention. *Adv. Nutr.* **14**(3), 555–569. 10.1016/j.advnut.2023.03.002 (2023). 36906146 10.1016/j.advnut.2023.03.002 PMC10201681

[7] Sádovská, G. & Wolf, G. Enthalpy of dissolution and thermal dehydration of calcium oxalate hydrates. *J. Therm. Anal. Calorim.* **119**, 2063–2068. 10.1007/s10973-014-4350-x (2015).

[8] Durdaği, S. P., Al- Jalawee, A. H. H., Yalçin, P., Bozkurt, A. S. & Salcan, S. Morphological characterization and phase determination of kidney stones using X-Ray diffractometer and scanning electron microscopy. *Chin. J. Phys.* **83**, 379–388. 10.1016/j.cjph.2022.08.025 (2023).

[9] İbiş-Özdemir, F. et al. Crystal growth of calcium oxalate mono- and dihydrate under laminar flow in microfluidic devices. *CrystEngComm* **27**, 337–346. 10.1039/D4CE01038H (2025).

[10] Goldberga, I. et al. First direct insight into the local environment and dynamics of water molecules in the whewellite mineral phase: mechanochemical isotopic enrichment and High-Resolution 17O and 2H NMR analyses. *J. Phys. Chem. C*. **126**, 12044–12059. 10.1021/acs.jpcc.2c02070 (2022). (2022). 10.1021/acs.jpcc.2c02070 PMC9340807 35928237

[11] Valido, I. H. et al. Discriminating the origin of calcium oxalate monohydrate formation in kidney stones via synchrotron microdiffraction. *Analyst* **147**, 349. 10.1039/d1an01703a (2022). 34935777 10.1039/d1an01703a

[12] Neira-Carrillo, A., Arce, T. Z. & Yazdani-Pedram, M. Controlled calcium oxalate crystals obtained by electrocrystallization on electrospun polycaprolactone fibers loaded with zarzaparrilla (*Herreria stellata*). *ACS Omega* **9**, 17045–17053. 10.1021/acsomega.3c08736 (2024). 38645337 10.1021/acsomega.3c08736 PMC11024939

[13] Cloutier, J., Villa, L., Traxer, O. & Daudon, M. Kidney stone analysis: “Give me your stone, i will tell you who you are!. *World J. Urol.* **33**, 157–169. 10.1007/s00345-014-1444-9 (2014). 25465911 10.1007/s00345-014-1444-9 PMC4308647

[14] Yehia, N. S., El-Sherfy, S. S. & Salam, R. A. Kinetics of dissolution of calcium oxalate monohydrate in the presence of hydrothiazide and Furosemide. *Chem Process. Eng. Res* **20** (2014).

[15] Liuab, N. et al. Shape and structure controlling of calcium oxalate crystals by a combination of additives in the process of biomineralization. *RSC Adv.* **8**, 11014–11020. 10.1039/C8RA00661J (2018). 35541543 10.1039/c8ra00661j PMC9078945

[16] Su, Y. et al. A molecular understanding of citrate adsorption on calcium oxalate polyhydrates. *Phys. Chem. Chem. Phys.* **25**, 12148–12156. 10.1039/D2CP04451J (2023). 37070707 10.1039/d2cp04451j

[17] Shanthil, M., Sandeep, K. & Sajith, P. K. Cooperative effects of Na^+^ and citrates on the dissolution of calcium oxalate crystals. *Phys. Chem. Chem. Phys.* **22**(8), 4788–4792. 10.1039/C9CP06499K (2020). 32068201 10.1039/c9cp06499k

[18] Peerapen, P., Putpeerawit, P., Boonmark, W. & Thongboonkerd, V. Resveratrol inhibits calcium oxalate crystal growth, reduces adhesion to renal cells and induces crystal internalization into the cells, but promotes crystal aggregation. *Curr. Res. Food Sci.* **8**, 100740. 10.1016/j.crfs.2024.100740 (2024). 38694557 10.1016/j.crfs.2024.100740 PMC11061250

[19] Kanlaya, R., Kuljiratansiri, R., Peerapen, P. & Thongboonkerd, V. The inhibitory effects of epigallocatechin-3-gallate on calcium oxalate onohydrate crystal growth, aggregation and crystal-cell adhesion. *Biomed. Pharmacother.* **170**, 115988. 10.1016/j.biopha.2023.115988 (2024). 38061137 10.1016/j.biopha.2023.115988

[20] Chaiyarit, S., Phuangkham, S. & Thongboonkerd, V. Quercetin inhibits calcium oxalate crystallization and growth but promotes crystal aggregation and invasion. *Curr. Res. Food Sci.* **8**, 100650. 10.1016/j.crfs.2023.100650 (2024). 38145155 10.1016/j.crfs.2023.100650 PMC10733680

[21] Chen, W. et al. Aqueous solubility, complex formation, and induction time of calcium oxalate monohydrate in the presence of natural organic small molecules. *J. Chem. Eng. Data*10.1021/acs.jced.4c00485 (2024).

[22] Yehia, N. S., Kashar, T. E., Abdaziz, A. M. A. & Kashkoush, S. G. Effect of some organic substances on kinetics of crystallization of calcium oxalate monohydrate and on some bacteria in urine. *Egypt. J. Chem.* **65**, 97–104. 10.21608/ejchem.2021.83412.4102 (2022).

[23] Yehia, N. S., Issa, F. A. & Kashcoush, S. G. Kinetic of dissolution of hydroxyapatite crystals in the presence of extracts of some medical plants. *Nat. Sci.* **15**, 12–31. 10.7537/marsnsj150317.03 (2017).

[24] Yehia, N. S. & Saleh, D. I. Influence of some amino acids on the mechanism of dissolution of calcium oxalate monohydrate crystals. *J. Am. Sci.* **10**, 182–190. 10.7537/marsjas100514.24 (2014).

[25] Kim, D. et al. Bio-inspired multifunctional disruptors of calcium oxalate crystallization. *Nat. Commun.* **16**, 5229. 10.1038/s41467-025-60320-4 (2025). 40473620 10.1038/s41467-025-60320-4 PMC12141576

[26] Werner, H. et al. Calcium oxalate crystallization: Influence of pH, energy input, and supersaturation ratio on the synthesis of artificial kidney stones. *ACS Omega* **6**, 26566–26574. 10.1021/acsomega.1c03938 (2021). 34661011 10.1021/acsomega.1c03938 PMC8515601

[27] Yehia, N. S., Ali, M. M., Kandil, K. M. & El-Maadaw, M. M. Effects of some parameters affecting the crystallization rate of calcium sulfate dihydrate in sodium chloride solution. *J. Am. Sci.* **7**, 635–644. 10.7537/marsjas070611.105 (2011).

[28] Mistry, S. et al. A novel, multi-barrier, drug eluting calcium sulfate/biphasic calcium phosphate biodegradable composite bone cement for treatment of experimental MRSA osteomyelitis in rabbit model. *J. Control. Release* **239**, 169–181. 10.1016/j.jconrel.2016.08.014 (2016). 27582374 10.1016/j.jconrel.2016.08.014

[29] White, R. L. Infrared spectroscopic investigations of calcium oxalate monohydrate (whewellite) dehydration/rehydration. *Minerals* **13**, 783. 10.3390/min13060783 (2023).

[30] Goher, S. S. et al. Electrospun tamarindus indica-loaded antimicrobial PMMA/cellulose acetate/PEO nanofibrous scaffolds for accelerated wound healing: In-vitro and in-vivo assessment. *Int. J. Biol. Macromol.* **258**, 128793. 10.1016/j.ijbiomac.2023.12879 (2024). 38134993 10.1016/j.ijbiomac.2023.128793

[31] Plaskova, A. & Mlcek, J. New insights of the application of water or ethanol-water plant extract rich in active compounds in food. *Front. Nutr.* **10**, 111876. 10.3389/fnut.2023.111876 (2023). 10.3389/fnut.2023.1118761 PMC10086256 37057062

[32] Amr, A. et al. UPLC-MS/MS analysis of naturally derived apis mellifera products and their promising effects against cadmium-induced adverse effects in female rats. *Nutrients* **15**, 119–136. 10.3390/nu15010119 (2023). 10.3390/nu15010119 PMC9823550 36615776

[33] Zoupanou, N. et al. Spectroscopic characterization using 1H and 13C nuclear magnetic resonance and computational analysis of the complex of donepezil with 2,6-Methyl-β-Cyclodextrin and hydroxy propyl methyl cellulose. *Molecules* **30**(5), 1169. 10.3390/molecules30051169 (2025). 40076392 10.3390/molecules30051169 PMC11902010

[34] Mulard, E. D., Gilard, V., Balayssac, S. & Rautureau, G. J. P. Quantitative nuclear magnetic resonance for small biological molecules in complex mixtures: Practical guidelines and key considerations for non-specialists. *Molecules* **30**(8), 1838. 10.3390/molecules30081838 (2025). 40333863 10.3390/molecules30081838 PMC12029823

[35] El-Wahed, A. A. A. et al. Sidr honeys physical and chemical characterization, a comprehensive approach through LC-MS/MS, NMR, and GC-MS analysis. *Separations* **10**, 372–391. 10.3390/separations10070372 (2023).

[36] Marafatto, F. F., Lanson, B. & Peñam, J. Crystal growth and aggregation in suspensions of δ-MnO_2_ nanoparticles: Implications for surface reactivity. *Environ. Sci.: Nano* **5**, 497–508. 10.1039/C7EN00817A (2018).

[37] Saad, M. A. et al. Chemically functionalized AlN quantum dots for effective sensing and removal of methanol, ethanol, and formaldehyde: A first principle study. *Chem. Phys.* **8**, 100620. 10.1016/j.chphi.2024.100620 (2024).

[38] Sakr, M. A. S. et al. Two-dimensional ZnS Quantum dots for gas sensors: Electronic and adsorption properties. *J. Electron. Mater.* **52**, 1–12. 10.1007/s11664-023-10455-1 (2023).

[39] Abdelsalam, H. et al. Highly efficient spin field-effect transistor based on nanographene and hBN heterostructures: spintronic and quantum transport properties. *Chin. J. Phys.* **90**, 237–251. 10.1016/j.cjph.2024.05.012 (2024).

[40] Spiegel, M. Current trends in computational quantum chemistry studies on antioxidant radical scavenging activity. *J. Chem. Inf. Model.* **62**, 2639–2658. 10.1021/acs.jcim.2c00104 (2022). 35436117 10.1021/acs.jcim.2c00104 PMC9198981

[41] Chan, B. Optimal small basis set and geometric counterpoise correction for DFT computations. *J. Chem. Theory Comput.* **19**, 3958–3965. 10.1021/acs.jctc.3c00298 (2023). 37288982 10.1021/acs.jctc.3c00298

[42] Pitman, S. J. et al. Benchmarking basis sets for density functional theory thermochemistry calculations: why unpolarized basis sets and the polarized 6-311G family should be avoided. *J. Phys. Chem. A*. **127**, 10295–10306. 10.1021/acs.jpca.4c00283 (2023). 37982604 10.1021/acs.jpca.3c05573

[43] Khaleghian, M. & Azarakhshi, F. Theoretical comparison of thermodynamic parameters, NMR analysis, electronic properties of Boron nitride and aluminum nitride nanotubes. *Int. J. Nano Dimens*. **10**, 105–113 (2019). http://creativecommons.org/licenses/by/4.0/

[44] Yang, Y. & Ostadhosseini, N. A theoretical investigation on the mercaptopurine drug interaction with Boron nitride nanocage: solvent and density functional effect. *Phys. E*. **125**, 114337. 10.1016/j.physe.2020.114337 (2021).

[45] Ge, Y. et al. Unraveling the intrinsic magnetic property of triangular zigzag edge bilayer graphene nanoflakes: A first-principles theoretical study. *Chem. Phys. Lett.* **730**, 326–331. 10.1016/j.cplett.2019.06.033 (2019).

[46] Abdelsalam, H. et al. Interaction of hydrated metals with chemically modified hexagonal Boron nitride quantum dots: wastewater treatment and water splitting. *Phys. Chem. Chem. Phys.* **22**, 2566–2579. 10.1039/C9CP06823F (2020). 31942882 10.1039/c9cp06823f

[47] Abdelsalam, H. et al. Two-dimensional Si2BN nanoflakes for efficient removal of heavy metals. *Chem. Phys. Lett.* **772**, 138568. 10.1016/j.cplett.2021.13856 (2021).

[48] Abdelsalam, H., Sakr, M. A. S., Saroka, V. A., Abd-Elkader, O. H. & Zhang, Q. Nanoroporous graphene quantum dots constructed from nanoribbon superlattices with controllable pore morphology and size for wastewater treatment. *Surfaces and Interfaces* **40**, 103109. 10.1016/j.surfin.2023.103109 (2023).

[49] Williamson, K. I., Herr, D. J. C. & Mo, Y. Toward tuning the bandgap in meta-substituted Fe-MOFs. *Mater. Adv.* **5**, 6842–6852. 10.1039/D4MA00512K (2024).

[50] Ho, K. V. et al. Identification and quantification of bioactive molecules inhibiting pro-inflammatory cytokine production in spent coffee grounds using metabolomics analyses. *Front. Pharmacol.* **11**, 229. 10.3389/fphar.2020.00229 (2020). 32210815 10.3389/fphar.2020.00229 PMC7073796

[51] Nemzer, B., Kalita, D. & Abshiru, N. Quantification of major bioactive constituents, antioxidant activity, and enzyme inhibitory effects of whole coffee cherries (*Coffea arabica*) and their extracts. *Molecules* **26**, 4306. 10.3390/molecules26144306 (2021). 34299581 10.3390/molecules26144306 PMC8305692

[52] Farag, M. A., Mohamed, T. A., El-Hawary, E. A. & Abdelwareth, A. Metabolite profiling of premium civet luwak bio-transformed coffee compared with conventional coffee types, as analyzed using chemometric tools. *Metabolites* **13**, 173. 10.3390/metabo13020173 (2023). 36837792 10.3390/metabo13020173 PMC9960232

[53] Correia, R. M. et al. Chemical profiles of robusta and arabica coffee by ESI(−)FT-ICR MS and ATR-FTIR: A quantitative approach. *Anal. Methods.* **8**, 7678–7688. 10.1039/C6AY02501C (2016).

[54] Szyszkiewicz-Warzecha, K. et al. The influence of chemical activity models on the description of ion transport through micro-structured cementitious materials. *Materials* **16**(3), 1116. 10.3390/ma16031116 (2023). 36770123 10.3390/ma16031116 PMC9920105

[55] Hamdona, S. K. & Al Hadad, U. A. Crystallization of calcium sulfate dihydrate in the presence of some metal ions. *J. Cryst. Growth* **299**(1), 146–151. 10.1016/j.jcrysgro.2006.11.139 (2007).

[56] Yang, J. M. & Compton, R. G. The dissolution and precipitation kinetics of solid particles: The influence of adsorption. *J. Solid State Electrochem.* **29**, 2101:210. 10.1007/s10008-025-06247-8 (2025).

[57] Hanaor, D. A. H., Ghadiri, M., Chrzanowski, W. & Gan, Y. Scalable surface area characterization by electrokinetic analysis of complex anion adsorption. *Langmuir* **30**, 15143–15152. 10.1021/la503581e (2014). 25495551 10.1021/la503581e

[58] Al‑Amiery, A., Isahak, W. N. R. W. & Al‑Azzawi, A. K. Multi-method evaluation of a 2-(1,3,4-thiadiazole-2-yl)pyrrolidine corrosion inhibitor for mild steel in HCl: Combining gravimetric, electrochemical, and DFT approaches. *Sci. Rep.* **13**, 9770. 10.1038/s41598-023-36252-8 (2023). 37328536 10.1038/s41598-023-36252-8 PMC10276050

[59] Chouchane, T. et al. Equilibrium, kinetics, and thermodynamics of batch adsorption of Mn(II) ions on blast furnace slag (BFS) and kaolin (KGA). *J. Eng. Appl. Sci.* **70**, 58. 10.1186/s44147-023-00218-4 (2023).

[60] Allaoui, I. et al. Adsorption equilibrium, kinetic, and thermodynamic studies on the removal of paracetamol from wastewater using natural and HDTMA-modified clay. *Desalin. Water Treat.* **318**, 100345. 10.1016/j.dwt.2024.100345 (2024).

[61] Acharya, A. et al. Quantum chemical calculations on calcium oxalate and dolichin A and their binding efficacy to lactoferrin: An in silico study using DFT, molecular docking, and molecular dynamics simulations. *AIMS Biophys.* **11**, 142–165. 10.3934/biophy.2024010 (2024).

[62] Quintero-Jaramillo, J., Carrero, J. A. & Sanabria-González, N. R. Caffeine adsorption on a thermally modified bentonite: Adsorbent characterization, experimental design, equilibrium and kinetics. *Colloids Interfaces* **8**(2), 26. 10.3390/colloids8020026 (2024).

[63] Li, S. et al. Dual mechanism of natural polyphenols on crystal whiskers formation on calcium oxalate monohydrate crystal surface. *Appl. Surf. Sci.* **592**, 153355. 10.1016/j.apsusc.2022.153355 (2022).

[64] Priadharshini, I. V. & Selvaraju, R. Growth and spectral characterization of calcium oxalate crystals. *Indian J. Sci. Technol.* **18**(23), 1862–1872. 10.17485/IJST/v18i23.877 (2025).

[65] Yan, Y. et al. The interactions of protein-calcium oxalate in crystallization process. *Chem. Eng. Sci.* **300**, 120649. 10.1016/j.ces.2024.120649 (2024).

[66] Xia, D. et al. Secondary products and molecular mechanism of calcium oxalate degradation by the strain Azospirillum sp. OX-1. *Sci. Rep.* **14**, 23506. 10.1038/s41598-024-74939-8 (2024). 39379461 10.1038/s41598-024-74939-8 PMC11461619

[67] Neira-Carrillo, A. et al. Electrocrystallization of calcium oxalate mediated by electrospun polymer fiber using poly(acrylic acid-co-4-styrene sulfonate). *Polymers* **17**(21), 2888. 10.3390/polym17212888 (2025). 41228648 10.3390/polym17212888 PMC12610098

