# Supplementary material for: Kinetics of dissolution and computational modeling of calcium oxalate monohydrate crystals in the presence of aqueous coffee bioactive extract compounds
Source: Sci Rep. 2026 Mar 23;16:9681. doi: 10.1038/s41598-026-40198-y (PMC13009524; doi:10.1038/s41598-026-40198-y)
Supplement: Supplementary file 1 — Supplementary Material 1 [file 41598_2026_40198_MOESM1_ESM.doc]

**Kinetics of Dissolution and Computational Modeling of Calcium Oxalate Monohydrate Crystals in the Presence of Aqueous Coffee Bioactive Extract Compounds**

**Eman T. Khattab^1^, Naema S. Yehia^1^, Mahmoud A. S. Sakr^2^, Hesham R. El-Seedi^1^, and Heba A. El-Shekheby^1,*^.**

^1^*Chemistry Department, Faculty of Science, Menoufia University, Shebin El-Kom, Egypt.*

^2^Center of Basic Science, Misr University for Science and Technology, 6^TH^ of October City, Egypt.

Corresponding author: Heba A. El-Shekheby, [heba.chem@yahoo.com](mailto:heba.chem@yahoo.com); [HEBA.alshakheby@science.menofia.edu.e*g*](mailto:HEBA.alshakheby@science.menofia.edu.eg)

**Supplementary Material**


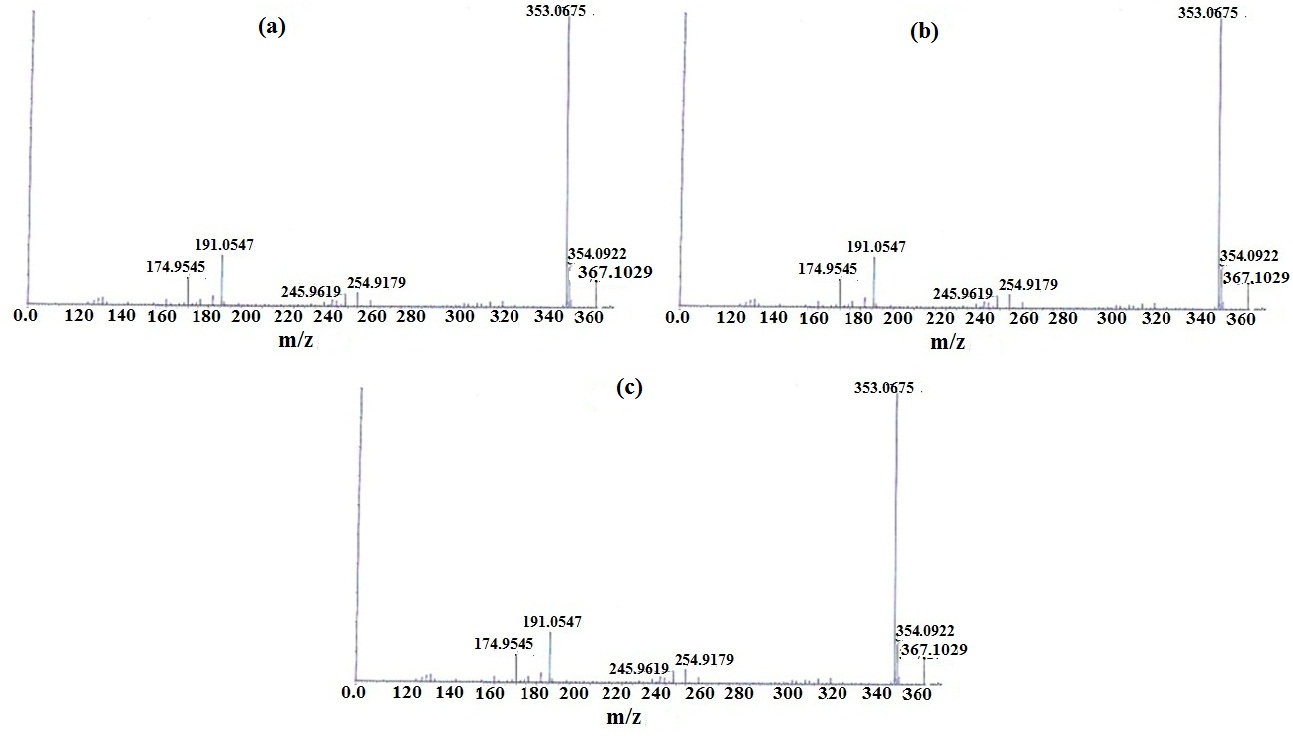


Fig. S1: LC-MS/MS chromatogram of: (a) chlorogenic acid, (b) 5-caffeoylquinic acid and (c) 4-caffeoylquinic acid identified from Cf in the negative ion mode.


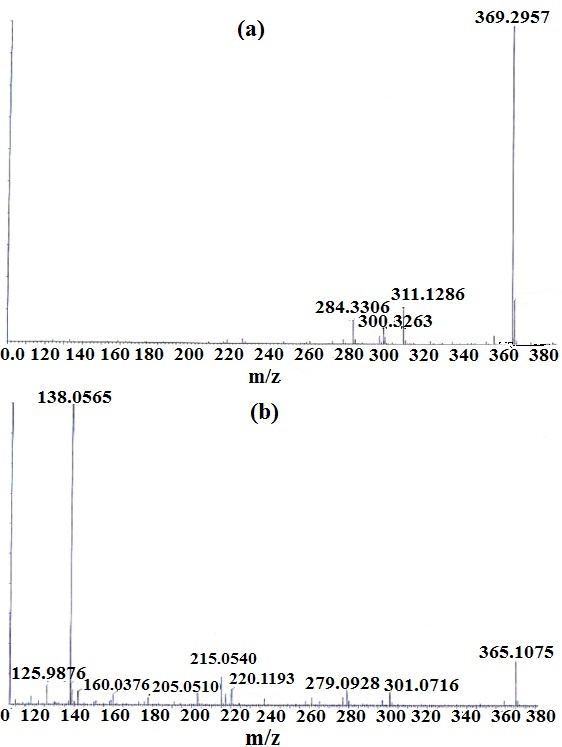


Fig. S2: LC-MS/MS chromatogram of (a) feruloylquinic acid (b) dimer of quinic acid identified from Cf in the positive ion mode.
